# The association of body image with quality of life, psychological assistance and social support in neurofibromatosis type 1 patients: a cross-sectional study

**DOI:** 10.1186/s13023-025-03729-w

**Published:** 2025-06-06

**Authors:** Daniel Muñoz, Mercè Jodar, Laia Valls, Albert Fornieles-Deu, Elisabeth Castellanos, Ignacio Blanco

**Affiliations:** 1 Clinical and Health Psychology Department, Universitat Autònoma de Barcelona (UAB), Bellaterra, Barcelona, Spain; 2 Clinical Genomics Group, CARE Program, Institut d’investigació Germans Trias i Pujol (IGTP), Badalona, Barcelona, Spain; 3 CSUR facomatosis, Servicio de Genética Clínica, Hospital Universitario Germans Trias i Pujol, Badalona, Barcelona, Spain; 4 Neurology Department, Hospital Universitari Parc Taulí, Institut d’Investigació i Innovació Parc Taulí (I3PT), Sabadell, Barcelona, Spain; 5 Centro de Investigación Biomédica en Red – Salud Mental (CIBERSAM), Instituto Carlos III, Barcelona, Barcelona, Spain; 6 Department of Psychobiology and Methodology of Health Sciences, Serra Húnter Fellow, Universitat Autònoma de Barcelona (UAB), Bellaterra, Barcelona, Spain; 7 Department of Surgery, Universitat Autònoma de Barcelona (UAB), Bellaterra, Barcelona, Spain

**Keywords:** Rare disease, Body image, Quality of life, Skin severity, Psychological assistance, Social support, Sources of support

## Abstract

**Background:**

Neurofibromatosis type 1 is a genetic disease with an autosomal dominant pattern. One of its clinical features is the presence of disfiguring neurofibromas. Most adults with Neurofibromatosis type 1 have visible neurofibromas depending on the severity of their skin related clinic that can affect their body image, and body image influencing psychological assistance and social support. This research explored Body image, the negative perception of the appearance of neurofibromas and skin severity in Neurofibromatosis type 1 patients; assessed its association with quality of life; and the role of social support and psychological assistance.

**Results:**

Two hundred five patients with Neurofibromatosis type 1 (16–74 years) were included in the study. They responded to questionnaires about their quality of life, body image and other sociodemographic data. Correlations and simple and multiple regressions were used to assess the relationships between variables. The results showed that body image problems increased if Neurofibromatosis type 1 patients were concerned about the aspects of their neurofibromas (B = 4.544; *p* < 0.001) and if they had severe skin conditions (B = 4.262; *p* < .001). Despite this, statistical analysis showed that only body image impairments reduced quality of life by 0.605 (*p* < 0.001), while skin severity and the negative perception of the appearance of neurofibromas were not clearly related. Patients with body image impairments are more likely to seek psychological assistance (ρ = 0.218; *p* < 0.01), but they are less likely to report having social support. The results also showed that when patients with Neurofibromatosis type 1 retrieved they have social support (ρ = -0.210, *p* < 0.01) or they inform doing psychological assistance (ρ = -0.238; *p* < 0.001), they have lower quality of life.

**Conclusion:**

Body image concerns, rather than skin severity, are a key feature for detecting quality of life impairments in these patients. When healthcare professionals detect body image impairments, it is crucial for them to collaborate with patients and either provide or refer them to psychological interventions. This approach helps improve social support, enabling patients to benefit from both their professional and personal environments.

**Supplementary Information:**

The online version contains supplementary material available at https://doi.org/10.1186/s13023-025-03729-w.

## Background

Neurofibromatosis type 1 (NF1) is a genetic disease with an autosomal dominant pattern. The incidence of this condition is 1/2000–3000, with 50% of individuals having de novo mutations [1].

The NF1 clinical manifestations consists of multiple café-au-lait macules (CALMs), which are present in 100% of children, skinfold freckling in non-sun-exposed areas (such as armpits or/and groins), Lisch nodules, scoliosis and disfiguring tumors of the nervous system (neurofibromas), specifically, plexiform neurofibromas (PN) that has a volume that increases faster than the body growth rate in some individuals [1–3]. Neurofibromas are complex tumors that arise constantly throughout life, especially in adolescence [4–6]. The majority of adults with NF1 have small neurofibromas throughout their body, and visible PN is present in 30% of patients [7].

It is important to acknowledge that skin is considered a vector of intimacy and sexuality, and when it is affected, it influences the perception of our own body [8]. The neurofibromas (PN or not) of NF1 adults may disfigure their body parts, and patients may experience body dissatisfaction because of this [7]. Therefore, in NF1, body image is affected [3, 7, 9]. Body image (BI) is a multidimensional construct that defines a variety of aspects related to an individual’s body appearance. It refers to how people perceive their own body and what attitudes and feelings people have toward their own body. It can be divided into four concepts: a) satisfaction with our body and their parts (body satisfaction), b) how one experiences and appraises their own body (body experience), c) being aware of our body (body awareness), and d) the boundaries we experience with our body parts (body boundaries). It develops during childhood and adolescence [7, 10]. Therefore, NF1 has been related to psychiatric and psychological signs such as depressive symptoms, dysfunctional coping and negative body image [6, 7, 11–13].

Despite the relevance of this topic, to date, there is no specific scale for evaluating body image in NF1 patients. The Body Image Scale (BIS) evaluates the BI for oncological patients with gynecological or breast cancer [14]. The Spanish version was adapted to the Spanish Body Image Scale (S-BIS). The scale consists of 10 items that measure different dimensions of the BI (affective, behavioral and cognitive). It has solid psychometric properties (α = 0.96) [15].

In addition to BI, patients with NF1 also report a negative impact on quality of life (QoL). Quality of life refers to the perception that individuals have about themselves and their role in life [16]. The degree and manner in which NF1 affects QoL vary greatly among individuals [11].

One of the factors that affects QoL in NF1 patients is the stigma caused by cutaneous neurofibromas [11, 12]. A greater number and wider distribution of cutaneous neurofibromas are strongly associated with poor skin-related QoL [13, 17]. Despite these findings, compelling evidence suggests that disease visibility alone does not predict general QoL [18]. In line with this, Granström et al. 2012 studied the relationship between disease visibility/severity and QoL through body image, proving that body experience mediates the link between disease visibility and poor QoL. According to Granström and colleagues adult NF1 patients expressing bodily insecurity/uneasiness and fewer feelings of attractiveness and self-confidence have a negative body image [7].

Building on this perspective, additional studies affirm that body image significantly affects QoL across all its subdomains [19]. Further investigation on the connections between BI, skin severity, neurofibromas and QoL will provide a better understanding of how these aspects are linked.

QoL and BI could improve with optimum interventions and social support [20]. Cognitive behavioral therapy (CBT), social skills training and psychoeducational interventions have been shown to address individuals’ negative self-perceptions and improve their psychosocial functioning [9, 13, 21, 22]. Moreover, the presence of friends, family or attempting a support group is of great importance. These relationships constitute social networks and provide experiences that may help adults overcome challenges associated with NF1 [20]. Patients actively strive to enhance their social support by employing adaptive strategies to better cope with their situation. One such strategy is positive reframing, where people emphasize the positive aspects of their lives, placing greater emphasis on their social support and evaluating it more positively [23]. Although body image is linked to psychological stress and social burden, few studies have examined BI in NF1 patients considering the evaluation of the social support and psychological assistance they need [7].

It is reasonable to hypothesize that the negative perception of the appearance of neurofibromas will correlate with increased concerns about body image (BI), and these impairments will have a more significant impact on the quality of life (QoL) of patients with neurofibromatosis type 1 (NF1) than solely evaluating the severity of skin manifestations. Additionally, due to this association, they will report increased psychological assistance and more social support. Therefore, this study aimed to explore the BI, the negative perception of the appearance of neurofibromas and the clinical skin severity of NF1 patients; assess each of these in association with quality of life; and to explore the relevance of social support and psychological assistance among NF1 patients dealing with BI concerns.

## Methods

A sample of 205 patients with NF1 who attended the Spanish National Reference Center for Neurocutaneous Syndrome-Phacomatosis (CSUR) of the Germans Trias and Pujol Hospital in the province of Barcelona were included in the study. The study was approved by the Ethics Research Committee of the Germans Trias i Pujol Hospital and was conducted in accordance with the latest version of the ethical principles of the Declaration of Helsinki.

**Fig. 1:**
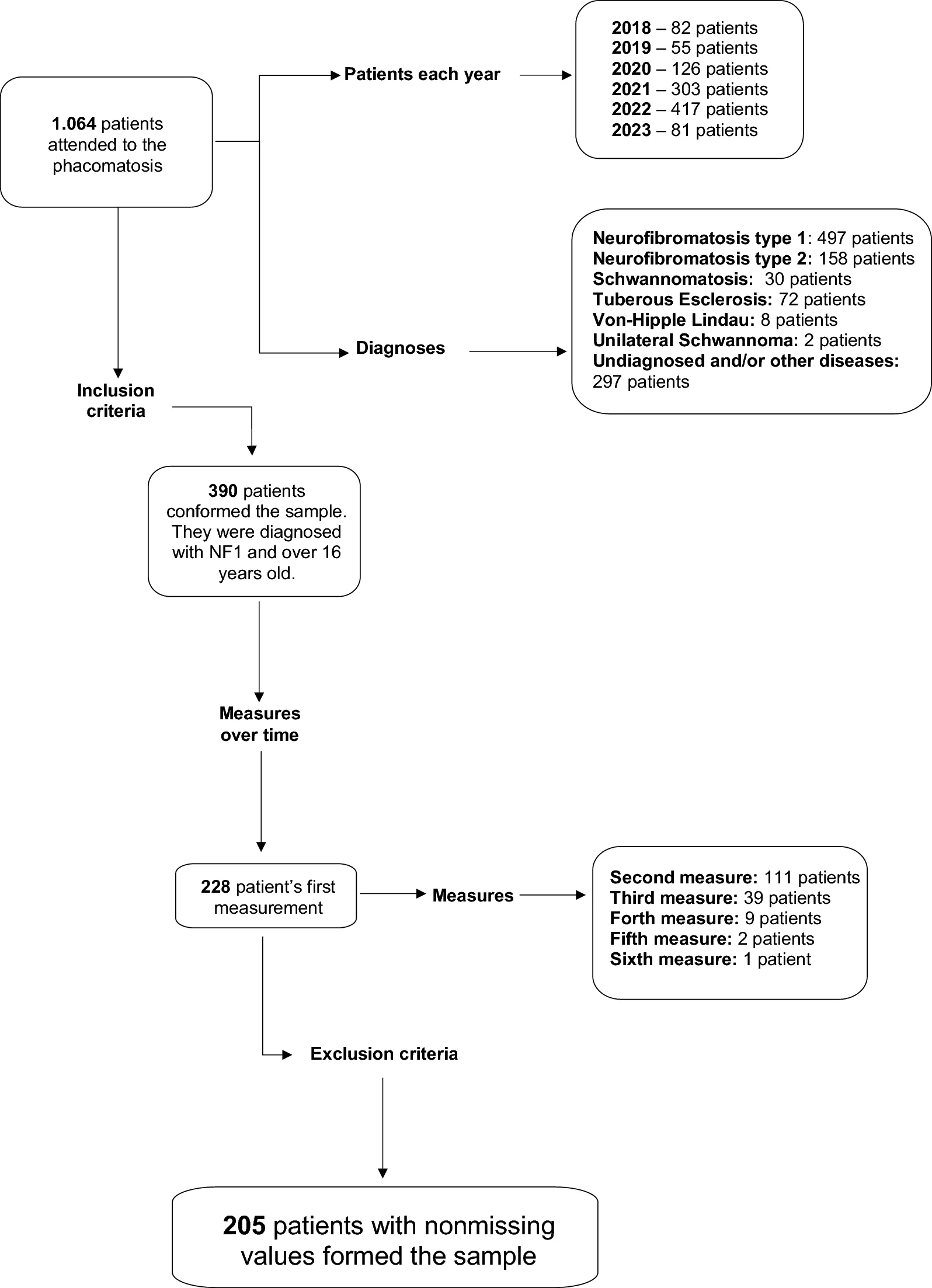
Flow diagram illustrating the sample process applying the inclusion and exclusion criteria

The study has a cross-sectional design. The inclusion criteria involved NF1 patients aged 16 years and older who were capable of responding to the survey questions. According to these criteria, we included all patients who visited the unit between February 2018 and May 2023 and agreed to participate in the study (Fig. 1). To ensure bias control, patients with missing data were excluded from the sample [24, 25].

All participants attended the Phacomatosis Unit of the Germans Trias and Pujol Hospital on scheduled dates to follow-up their health condition. Informed consent was provided by the participants prior to the assessment. A trained psychologist administered all the questionnaires.

### Assessment

**Sociodemographic data:** Patients provided their gender, age, education, marital status and employment status. Age was divided into five age groups: adolescents (16–18 years), young adults (19–24), adults (25–44 years), middle-aged individuals (45–64 years) and aged individuals (65–80 years).

**Psychological assistance:** Was evaluated with a dichotomized question asking if they were receiving psychological assistance at the time of evaluation. The duration of the therapy, any previous psychological assessment or the perceived effectiveness were not considered.

**Social Support:** Social support was assessed with a dichotomized question asking whether the participants had social support. Patients who answered “Yes” were prompted to select their sources of support from six options: social networks, institutions, others, family, friends and partner. They can choose more than one source.

**Modified Spanish body image scale:** The modified version of the S-BIS used in the study was adjusted from the original S-BIS. S-BIS is composed of 10 items evaluating various aspects of BI in cancer patients, including affective (e.g. feeling self-conscious), behavioral (e.g. avoiding looking at one’s body) and cognitive (e.g. dissatisfaction with appearance) dimensions. Each item is rated on a four-point scale from 0 (not at all) to 3 (very much). The total score, ranges from 0 to 30, is calculated adding all items, with higher scores indicating higher BI impairments [15]. We removed items 8 and 10 because the NF1 clinic does not involve feeling the body less complete or having scars (Supplementary material 1).

**Neurofibromas question (Ad. Hoc):** We included a question referring to patient satisfaction with the appearance of their neurofibromas: *“Have you felt unsatisfied with the way your neurofibromas look like”.* It follows a Likert structure similar to the S-BIS, and their scores vary from 0 (not at all) to 3 (a lot). The S-BIS scale captures cognitive aspects of body image but does not specifically address neurofibromas, which we considered a key feature of the NF1 skin manifestations. Thus, we included this additional question to address the impact of neurofibromas on body image directly.

**Skin severity:** Skin severity index values ranging from 1 to 3 according to the clinical guidelines of the hospital. These grades were defined by the qualitative impact and severity of the clinical characteristics of NF1 on these patients. The clinicians of the Phacomatosis unit (dermatologist, geneticists, neurologists and genetic counselors) combine their opinions to classify them into one of these grades.

- Grade 1 or mild involvement: indicates the presence of some features of the disease, such as a café au lait spot color and few neurofibromas.
- Grade 2 or moderate involvement: There was no significant compromise to the patient’s health; there were café au lait-colored spots and a modest number of cutaneous and/or subcutaneous neurofibromas.
- Grade 3 or severe affectation: is characterized by the presence of plexiform neurofibromas and/or a significant number of cutaneous and subcutaneous neurofibromas throughout the body. These skin manifestations affect the patient’s health.

**QoL** was assessed with the EuroQol Quality of Life Scale (EQ-5D) [26, 27]. It consists of a descriptive system in which the individual assesses their health status in five dimensions: mobility, personal care, daily activities, pain/discomfort and anxiety/depression. Each dimension is valued at three levels of severity: without problems, with problems or moderate problems and with serious problems. The scale also includes a vertical visual analog scale (EQ-VAS), where individuals must assess their global health status, from the worst imaginable health state (0) to the best state of health imaginable (100) [28].

### Statistical analysis

The statistical analysis (descriptive, bivariate and multivariate) was conducted using R version 4.2.2. Shapiro–Wilk tests were performed for each variable to evaluate the data distribution. We included means and standard deviations for quantitative variables and frequencies and percentages for qualitative variables, and we used the Mann‒Whitney U test for quantitative variables and the chi2 test for qualitative variables with gender, psychological assistance and social support. We also analyzed the S-BIS modified with the exploratory factor analysis (EFA) method [29–31]

We retrieved Spearman correlations and simple regressions with the independent variables (S-BIS modified and Neurofibromas question Ad Hoc) and the most significant covariables (social support and psychological assistance) to test their relationships with the other variables and determine whether social support and psychological assistance are associated with BI.

The multivariate analysis consisted of multiple regressions with a stepwise method to test whether QoL was related to BI. We made two blocks: the first one contained all sociodemographic variables (gender, age, education, marital status and employment), and the second one included the independent variables of our study (S-BIS-modified and Neurofibromas Question Ad Hoc).

The researchers include the variables following the adjusted R-squared statistic of the goodness of fit method [32]. The criteria fixed to exclude a variable from the model were set to *p* =  > 0.05 [33]

## Results

### Description of the sample and S-BIS modified

The sample consisted of 205 patients with NF1 diagnoses, above 16 years old and with no missing data (Fig. 1). Presented 93 male patients and 112 female patients. The majority of the participants were young adults or adults (19–44), were single, had studied until high school, were employed and presented social support. Their family mostly gave this support. They did not receive psychological assistance, and their skin severity index was low. The average age was 33 years, with a good QoL (0.77/71.6) and low body image-related problems (5.13). A large part of the sample reported no problems at all with their tumors (Table 1).

**Table 1:** Sample description—sociodemographic, dependent and independent variables

Variables	Categories/ Ranges	Total (n = 205)		Male (n = 93/ % = 45.37)		Female (n = 112 / % = 54.63)		W (p) / X^2^ (p)
		N (%)	M (SD)	N (%)	M (SD)	N (%)	M (SD)	
Age	16–74	–	33.67 (13.81)	–	31.47 (13.73)	–	35.5 (13.68)	4371 (< 0.001)***
Age groups	Adolescent (16–18)	7 (3.41)	–	2 (2.15)	–	5 (4.46)	–	14.01 (0.007)**
	Young Adult (19–24)	74 (36.10)	–	46 (49.46)	–	28 (25.00)	–	
	Adult (25–44)	73 (35.61)	–	25 (26.88)	–	48 (42.86)	–	
	Middle aged (45–64)	44 (21.46)	–	18 (19.35)	–	26 (23.21)	–	
	Aged (65–80)	7 (3.41)	–	2 (2.15)	–	5 (4.46)	–	
Marital status	Married	55 (26.83)	–	15 (16.13)	–	40 (35.71)	–	13.58 (0.003)**
	Single	141 (68.78)	–	76 (81.72)	–	65 (58.04)	–	
	Divorced	8 (3.90)	–	2 (2.15)	–	6 (5.36)	–	
	Widowed	1 (0.49)	–	0 (0)	–	1 (0.89)	–	
Education	No-studies	4 (1.95)	–	2 (2.15)	–	2 (1.79)	–	7.61 (0.107)
	Primary school	27 (13.17)	–	15 (16.13)	–	12 (10.71)	–	
	Middle school	38 (18.54)	–	21 (22.58)	–	17 (15.18)	–	
	High school	78 (38.05)	–	37 (39.78)	–	41 (36.61)	–	
	University	58 (28.29)	–	18 (19.35)	–	40 (35.71)	–	
Employment	Employed	107 (52.19)	–	48 (51.61)	–	59 (52.68)	–	0.341 (0.952)
	Unemployed	83 (40.49)	–	39 (41.93)	–	44 (39.29)	–	
	Paid leave	12 (5.85)	–	5 (5.38)	–	7 (6.25)	–	
	Retired	3 (1.46)	–	1 (1.07)	–	2 (1.79)	–	
Skin severity index^a^	1–3	–	1.62 (0.77)	–	1.63 (0.75)	–-	1.61 (0.80)	4371 (< 0.001)***
	Low severity	115 (56.10)	–	49 (52.69)	–	66 (58.93)	–	2.57 (0.277)
	Medium severity	53 (25.85)	–	29 (31.18)	–	24 (21.43)	–	
	High severity	37 (18.05)	–	15 (16.13)	–	22 (19.64)	–	
Psychological assistance	Yes	62 (30.24)	–	22 (23.65)	–	40 (35.71)	–	2.95 (0.086)
	No	143 (69.76)	–	71 (76.34)	–	72 (64.29)	–	
Social support	Yes	158 (77.07)	–	66 (74.19)	–	89 (79.46)	–	0.53 (0.467)
	No	47 (22.93)	–	27 (25.81)	–	23 (20.54)	–	
Sources of support	Social networks	3 (1.46)	–	2 (2.15)	–	1 (0.89)	–	0.03 (0.871)
	Institutions	9 (4.39)	–	4 (4.30)	–	5 (4.46)	–	< 0.001 (1)
	Others	9 (4.39)	–	4 (4.30)	–	5 (4.46)	–	< 0.001 (1)
	Family	145 (70.73)	–	60 (64.52)	–	85 (75.89)	–	2.65 (0.103)
	Friends	79 (30.24)	–	33 (35.48)	–	46 (41.07)	–	0.45 (0.500)
	Partner	39 (19.02)	–	18 (46.15)	–	21 (53.85)	–	< 0.001 (1)
EuroQoL (EQ-5D)	0.00–1.00	–	0.77 (0.22)	–	0.82 (0.22)	–	0.73 (0.22)	4278 (< 0.001)***
EuroQol Visual Scale (EQ-VAS)	0–100	–	71.6 (26.00)	–	76.57 (24.15)	–	67.48 (26.85)	4005 (< 0.001)***
S-BIS modified	0–24	–	5.13 (6.25)	–	3.63 (6.25)	–	6.36 (6.67)	1485 (< 0.001)***
Neurofibromas (Ad Hoc) ^b^	0–3	–	0.95 (1.14)	–	0.75 (1.04)	–	1.11 (1.20)	820 (< 0.001)***
	Not at all	103 (50.24)	–	53 (56.99)	–	50 (44.64)	–	5.03 (0.169)
	A little	45 (21.95)	–	21 (22.58)	–	24 (21.43)	–	
	Quite a bit	22 (10.73)	–	8 (8.60)	–	14 (12.50)	–	
	Very much	35 (17.07)	–	11 (11.83)	–	24 (21.43)	–	

Total data and segmentation by gender

N, number of subjects; %, percentages; M, means; SD, standard deviations; W, Wilcoxon tests and X^2^, chi-squared test for non-parametric variables; p, Significance level: < 0.05 (*), < 0.01 (**), < 0.001 (***). ^a^ Skin Severity was considered numerical and categorical. ^b^ Neurofibromas (Ad Hoc) was considered numerical and categorical

We tested the psychometric properties of the S-BIS modified and their relationships with the neurofibromatosis question Ad Hoc. The scale presented good validity and reliability requirements. The Cronbach’s alpha of the scale was 0.94 and presented a one-factor structure, similar to the original S-BIS version; the proportion of variance was 68%. The item total, inter item and item-rest correlations were above 0.4, which indicates that each item contributes to the internal consistency of the scale. The alpha if an item is dropped remains the same. Furthermore, it correlated with the EQ-5D and EQ-5D VAS scores (-0.52/-0.53). The neurofibromas assessed by the Ad Hoc test correlate with the modified S-BIS 0.83 and with EQ-5D and the EQ-VAS (-0.37/-0.41), respectively (Supplementary materials 2 and 3).

#### Body image, negative perception of the neurofibromas and skin severity index

The simple regression results show that a higher score on the neurofibromas question Ad Hoc increased the S-BIS score by 4.544 (< 0.001).

The S-BIS-modified and neurofibromas questions were positively correlated with gender and age. The skin severity index increased by 2.009 for the modified S-BIS, and individuals with a high severity score were 4.262 more likely to have higher scores than those with a low severity score, with both relationships being significant at < 0.001.

The findings from the regression analysis concerning the question about neurofibromas suggested that the skin severity index increased by 0.464. High-severity-rated individuals are more prone to neurofibroma appearance problems than low-severity-rated individuals are, with both relationships being significant at < 0.001 (Table 2).

**Table 2:** Simple regressions with the independent variables and the sociodemographic data

Variables ^a^	Categories^b^	Total (n = 205)						
		B	β	SE (B)	t value	Pr ( >|t|)	95% CI (B)	
S-BIS modified								
Gender	Male/ female	2.722	0.217	0.857	3.175	0.002**	1.032	4.413
Age	16–74	0.100	0.221	0.031	3.225	0.001**	0.039	0.161
Age Groups	Adolescent/ young adult	− 2.263	− 0.174	0.897	− 2.524	0.012*	− 4.031	− 0.495
	Adolescent/ middle aged	3.260	0.215	1.040	3.133	0.002**	1.208	5.311
Skin Severity Index	–	2.009	0.246	0.568	3.537	< 0.001***	0.909	3.072
	Low severity/ high severity	4.262	0.245	1.167	3.651	< 0.001***	1.800	6.147
Neurofibromas (Ad Hoc)	–	4.544	0.828	0.216	21.046	< 0.001 ***	4.118	4.969
*Neurofibromas (*Ad Hoc*)*								
Gender	Male/ female	0.354	0.155	0.158	2.241	0.026*	0.043	0.666
Age	16–74	0.022	0.267	0.006	3.956	< 0.001***	0.011	0.033
Age Groups	Adolescent/ young adult	− 0.550	− 0.232	0.161	− 3.41	< 0.001***	− 0.869	-0.232
	Adolescent/middle aged	0.734	0.265	0.187	3.921	< 0.001***	0.365	1.103
Employment	Unemployed/ paid leave	0.854	0.176	0.334	2.554	0.011*	0.195	1.513
Skin severity index	–	0.464	0.316	0.098	4.744	< 0.001***	0.271	0.657
	Low severity/ high severity	0.857	0.290	0.198	4.321	< 0.001***	0.466	1.248

B, regression coefficient; β, standardized regression coefficient; SE, standard error of B; t value of B; Pr ( >|t|), Significance level: < 0.05 (*), < 0.01 (**), < 0.001 (***); 95% CI, 95% confidence interval of B; ^a^ The variables selected correspond to those that significantly correlated with both variables; ^b^ We created dummy variables for those qualitative variables with more than two categories

### Quality of life

The EQ-5D regressions include gender, age, marital status, education, employment and skin severity indices in the statistical model. Patients whose skin severity was moderate were 0.082 more likely to have higher scores on the EQ-5D than those whose skin severity was low, but there were no significant differences between patients with higher severity scores and those with higher severity scores on the EQ-5D. The modified S-BIS is included because it is significant (< 0.001). High scores on the modified S-BIS are related to a 0.21 decrease in the EQ-5D score. The neurofibromas question Ad Hoc was not significantly related to the EQ-5D score.

The EQ-VAS, includes gender, age and education in its statistical model. The S-BIS-modified scale is included because it is significant (< 0.001). High scores on the modified S-BIS (2.599) decrease scores on the EQ-VAS, with this finding being significant at < 0.001. The neurofibromas question Ad Hoc was not significantly related to the EQ-VAS score (Table 3).

**Table 3:** Multiple regressions of the EQ-5D and EQ-VAS scores with the independent variables: adjusted for covariables

	Variables^a^	Categories^b^	Total (n = 205)						
			B	β	SE (B)	t value	Pr( >|t|)	95% CI (B)	
*EQ-5D*									
Covariables	Gender	Male/ female	− 0.043	− 0.096	0.062	− 1.558	0.121	− 0.217	0.025
	Age	–	− 0.003	− 0.201	0.085	− 2.371	0.019*	− 0.367	− 0.035
	Marital Status	Single/ married	0.114	0.229	0.082	2.797	0.006**	0.069	0.389
		Single/ divorced	0.173	0.152	0.065	2.343	0.020*	0.025	0.278
	Education	No-studies/ middle school	0.042	0.092	0.060	1.536	0.126	− 0.025	0.210
	Employment	Unemployed/ paid leave	− 0.103	− 0.110	0.061	− 1.806	0.072	− 0.229	0.009
	Skin Severity Index	Low severity/ medium severity	0.082	0.163	0.063	2.580	0.011*	0.039	0.286
		Low severity/ high severity	0.016	0.028	0.065	0.430	0.667	− 0.099	0.155
Independent variables	S-BIS modified	–	− 0.021	− 0.605	0.106	− 5.710	< 0.001***	− 0.813	− 0.397
	Neurofibromas (Ad Hoc)	–	0.033	0.170	0.108	1.579	0.116	-0.041	0.382
*EQ-VAS*									
Covariables	Gender	Male/ female	− 3.230	− 0.062	0.062	− 1.007	0.315	− 0.183	0.059
	Age Groups	Adolescent/ young adult	6.145	0.114	0.063	1.814	0.071	− 0.009	0.237
		Adolescent/ Aged	− 10.825	− 0.076	0.058	− 1.297	0.196	− 0.190	0.039
	Education	No-studies/ primary school	31.952	0.417	0.149	2.793	0.006**	0.124	0.709
		No-studies/ middle school	33.550	0.503	0.168	3.001	0.003**	0.174	0.831
		No-studies/ high school	42.264	0.791	0.206	3.842	< 0.001***	0.388	1.195
		No-studies/ University	42.661	0.741	0.192	3.861	< 0.001***	0.365	1.117
Independent variables	S-BIS modified	–	− 2.599	− 0.624	0.104	− 6.023	< 0.001***	− 0.827	− 0.421
	Neurofibromas (Ad Hoc)	–	4.370	0.191	0.104	1.846	0.066	-0.012	0.394

B, regression coefficient; β, standardized regression coefficient; SE, standard error of B; t value of B; Pr ( >|t|), Significance level: < 0.05 (*), < 0.01 (**), < 0.001 (***); 95% CI, 95% confidence interval of B; ^a^ The table shows the variables selected following the Stepwise method; ^b^ We created dummy variables for those qualitative variables with more than two categories

QoL is associated with the S-BIS-modified questionnaire and the Neurofibromas ad. Hoc question. Simple regressions show that each subscale of the EQ-5D is related to the modified S-BIS, all of which have a significance < 0.05. Daily activities, pain and anxiety/depression were significant at < 0.001. The Neurofibromas Questionnaire (Ad Hoc) is related to daily activities, pain and anxiety/depression, with a significance < 0.001. Self-care and mobility were not significantly related to the question (Supplementary material 4).

### Psychological assistance and social support

The correlations of psychological assistance show that it is directly associated with the skin severity index (0.159, < 0.05), especially for high-severity patients (0.188, < 0.01), and with social support (0.208, < 0.01), especially if support is given by family members (0.190, < 0.01), friends and others (< 0.05). Additionally, the modified S-BIS correlated directly with performing psychological assistance (0.218, < 0.01) but not with the neurofibromas question Ad Hoc. In contrast, the EQ-5D score is inversely associated with psychological assistance (− 0.238, < 0.01) (Table 4).

**Table 4:** Correlations with Psychological Assistance

		Psychological Assistance	
Variable	Categories	ρ	Pr( >|p|)
Gender	Male/ female	0.131	0.062
Age	–	− 0.112	0.111
Age groups	Adolescent/ young adult	0.102	0.145
	Adolescent/ adult	− 0.002	0.980
	Adolescent/ middle Aged	− 0.111	0.112
	Adolescent/ aged	− 0.007	0.922
Marital status	Single/ married	− 0.183	0.009**
	Single/ divorced	0.032	0.651
	Single/ Widowed	− 0.046	0.512
Education	No-studies/ primary school	0.026	0.709
	No-studies/ middle school	− 0.095	0.173
	No-studies/ hyigh school	− 0.057	0.420
	No-studies/ University	0.152	0.029*
Employment	Unemployed/ employed	− 0.135	0.053
	Unemployed/ paid leave	0.017	0.811
	Unemployed/ retired	− 0.080	0.253
Skin severity index	–	0.159	0.023*
	Low severity/ medium severity	− 0.025	0.722
	Low severity/ high severity	0.188	0.007**
Social support	No/ yes	0.208	0.003**
Sources of support	No / social networks	0.097	0.168
	No / institutions	0.066	0.345
	No / others	0.170	0.015*
	No / family	0.190	0.006**
	No / friends	0.155	0.026*
	No / partner	− 0.022	0.760
EQ-5D	–	− 0.238	< 0.001***
EQ-VAS	–	− 0.101	0.150
S-BIS modified	–	0.218	0.002**
Neurofibromas (Ad Hoc)	–	0.077	0.270

ρ, Spearman’s coefficient; Pr ( >|ρ|), Significance level: < 0.05 (*), < 0.01 (**), < 0.001 (***)

The correlations with social support and sources of support show that social support is directly associated with psychological assistance and with family, friends and partner support (< 0.01). The EQ-5D score was negatively correlated with social support (− 0.210, < 0.01). Sources of support from others, from family and from friends correlated positively with psychological assistance (< 0.05). The EQ-5D score was inversely associated with family support (− 0.169, < 0.05). The medium severity of skin severity was negatively associated with social support and family and friends support (< 0.05). Social support and sources of support were not correlated with S-BIS-modified or Neurofibromas Question Ad Hoc scores (Table 5 and Supplementary material 5).

**Table 5:** Correlations with social support

		Social support	
Variable	Categories	ρ	Pr ( >|p|)
Gender	Male/fdemale	0.062	0.374
Age	–	− 0.179	0.010*
Age Groups	Adolescent/ young adult	0.144	0.039*
	Adolescent/ adult	− 0.031	0.663
	Adolescent/ middle Aged	− 0.139	0.047*
	Adolescent/ aged	-0.089	0.204
Marital Status	Single/ married	− 0.115	0.101
	Single/ divorced	-0.010	0.888
	Single/ widowed	0.038	0.587
Education	No-studies/ primary school	0.075	0.284
	No-studies/ middle school	− 0.068	0.330
	No-studies/ high school	0.021	0.764
	No-studies/ University	0.008	0.913
Employment	Unemployed/ employed	− 0.150	0.032*
	Unemployed/ paid leave	0.136	0.052
	Unemployed/ retired	− 0.127	0.070
Skin Severity Index	-	− 0.094	0.180
	Low severity/ medium severity	− 0.155	0.026*
	Low severity/ high severity	0.015	0.836
Psychological Assistance	No/ yes	0.208	0.003**
Sources of Support	No / social Networks	0.066	0.344
	No / institutions	0.117	0.095
	No / others	0.117	0.095
	No / family	0.848	< 0.001***
	No / friends	0.432	< 0.001***
	No / partner	0.264	< 0.001***
EQ-5D	–	− 0.210	0.002**
EQ-VAS	–	− 0.086	0.223
S-BIS modified	–	0.119	0.089
Neurofibromas (Ad Hoc)	–	0.004	0.953

ρ, Spearman’s coefficient; Pr ( >|ρ|), Significance level: < 0.05 (*), < 0.01 (**), < 0.001 (***)

## Discussion

This research explored BI, the negative perception of the appearance of neurofibromas and clinical skin severity in NF1 patients; assessed its association with quality of life; and demonstrated the relevance of social support and psychological assistance among NF1 patients with BI concerns.

The vast majority of our NF1 cohort presented minor skin severity, only 18% of the individuals have high severity. Therefore, few of them reported negative perception of the appearance of neurofibromas, and they presented positive BI and QoL. For those who rated high skin severity, we noticed that they also had BI impairments and negative perception of the aspect of neurofibromas. When the skin is altered in NF1 patients, body image is disturbed [8]. Moreover, as we hypothesized, our results show that negative perception of the appearance of neurofibromas is an important factor in the increase in BI impairment in NF1 patients, especially considering that over 50% of our patients reported dissatisfaction with the appearance of their neurofibromas.

NF1 individuals from our CSUR had significantly lower QoL only if they had severe BI impairment, not if they reported negative perception of the appearance of neurofibromas. Previous studies have suggested that when cutaneous neurofibromas are numerous and extensively cover the body surface, individuals are more likely to experience a diminished QoL [13]. However, it is essential to note that the visibility of the disease itself does not reliably predict overall QoL [18]. We must consider the role of body image as a moderator in the relationship between disease visibility and QoL impairments [18].

Negative perception of the appearance of neurofibromas does not affect QoL in the same way as BI problems do. Accordingly, even though discontent with the appearance of neurofibromas plays an important role in BI, BI impairments do not merely derive from the negative perception the patient have about the appearance of their neurofibromas.

Therefore, instead of relying solely on the severity of skin manifestations to assess QoL, it is accurate to consider evaluating the overall concept of BI as a more meaningful indicator of overall QoL. Indeed, BI impairments affect each QoL domain evaluated by the EQ-5D and the EQ-VAS [19]. Modified S-BIS used in this study presented good validity and reliability requirements and correlated with the QoL test and the question referring to the neurofibromas appearance. Hence, it proves to be a reliable tool for assessing body image in NF1 patients.

Patients with NF1 and severe skin involvement, in our sample, correlated with increased body image concerns and negative perception of the appearance of neurofibromas. Various studies have reported that optimum psychological interventions could improve BI in these patients [12, 20, 21]. Consequently, it is reasonable that our patients reported having undergone or currently undergoing psychological assistance to address these issues.

Similarly, NF1 patients with poor QoL tend to report attending psychological assistance, and they also tend to inform that they have social support, especially from their family [11, 23]. The distinction between psychological assistance and social support becomes evident when considering that body image impairments do not seem to affect how NF1 patients perceive their social support. However, patients who report having received psychological assistance are more likely to report having social support networks, possibly because they learn coping strategies to acquire or maintain their social support. Therefore, psychological assistance appears to be an effective strategy for coping with body image impairments in NF1 patients.

### Limitations of the study

A comprehensive understanding of the aspects involved in therapy, such as alliance, expectations, therapist effects, cultural adaptation of evidence-based treatments, treatment differences, accessibility and adherence and competence, is needed to better identify whether individuals who are receiving psychological assistance can address body image impairments and diminish their effects on NF1 patients’ QoL [34]. In this regard, it is crucial to implement longitudinal measurements that consider the progression of psychological assistance over time.

Additionally, the assessment of social support extends beyond the mere identification of sources of support. To enhance the understanding of its implications, measurements should also evaluate how patients derive tangible benefits from these supportive networks [11].

## Conclusion

The findings of this study are particularly relevant to the minority population diagnosed with NF1, shedding light on the unique challenges they face and advancing research in the field of genetic disorders.

This study emphasizes the role of body image impairments as predictors of reduced quality of life (QoL) in NF1 patients. The Modified S-BIS questionnaire provides an effective tool to identify individuals at risk of QoL decline, enabling timely psychological intervention. Compared to skin severity, body image is a stronger indicator of QoL impairments. The Modified S-BIS directly measures body image issues without relying on clinical cut-off points, making it a valuable tool for ongoing patient monitoring. It also captures feelings of bodily insecurity—such as reduced attractiveness and self-confidence—which have been previously linked to negative body image in NF1 patients.

An increase in a patient’s body image impairment score over time could signal emerging QoL issues. Healthcare professionals should consider this metric in their clinical practice to shape effective interventions and improve the overall patient experience. Additionally, our findings support previous studies that highlight body image as a key factor in how NF1 patients perceive their bodies and how this perception influences their psychological well-being and QoL.

NF1 patients benefit from both positive social support and psychological assistance, particularly when facing QoL impairments. Notably, when patients seek psychological counseling, they often report having social support. However, a key difference arises when body image impairments increase. In these cases, patients may report receiving psychological support but not social support. Therefore, when healthcare professionals detect body image impairments in NF1 patients, it is crucial to guide them toward psychological therapies that also focus on enhancing social skills and providing psychoeducation for families and close social networks. This approach can help improve social support, ensuring that patients benefit from both professional and personal environments. Future research should explore the effects of psychological assistance and social support on NF1 patients with body image impairments.

## Supplementary Information

Supplementary material 1: The modified S-BIS.

Supplementary material 2: EFA analysis: S-BIS modified – Validity and Reliability Requirements; The Exploratory Factorial Analysis of the S-BIS modified scale.

Supplementary material 3: Correlations between independent and dependent variables; Correlations between independent and dependent variables

Supplementary material 4: Simple regressions with the independent variables and the EQ-5D subscales.; Simple regressions performed with the EQ-5D subscales

Supplementary material 5: Correlations with Sources of Support; Correlations with the sources of support measures in the study

## Data Availability

The data that support the findings of this study are available from Germans Trias i Pujol Hospital, but restrictions apply to the availability of these data, which were used under license for the current study and are not publicly available. However, the data are available from the authors upon reasonable request and with the permission of Germans Trias I Pujol Hospital.

## Abbreviations

NF1: Neurofibromatosis type 1
CALM’s: Café-au-lait macules
PN: Plexiform neurofibroma
BI: Body image
BIS: Body image scale
S-BIS: Spanish version of the body image scale
QoL: Quality of life
CBT: Cognitive behavioral therapy
CSUR: Centers, services and units of reference
EQ-5D: EuroQoL quality of life scale
EQ-VAS: EuroQoL quality of life visual analog scale
EFA: Exploratory factorial analysis
