# Supplementary material for: The association of body image with quality of life, psychological assistance and social support in neurofibromatosis type 1 patients: a cross-sectional study
Source: Orphanet J Rare Dis. 2025 Jun 6;20:284. doi: 10.1186/s13023-025-03729-w (PMC12143036; doi:10.1186/s13023-025-03729-w)
Supplement: Supplementary file 1 — Supplementary material 1: The modified S-BIS. [file 13023_2025_3729_MOESM1_ESM.pdf]

Additional File 1: The Modified S-BIS

CUESTIONARIO DE IMAGEN CORPORAL En este cuestionario se le preguntará cómo se siente en relación a su apariencia. Por favor, lea cuidadosamente cada ítem y marque con una X aquella respuesta que más se aproxime a la forma cómo se ha sentido durante **la semana pasada.**

Pregunta	En absoluto	Un poco	Bastante	Mucho
¿Se ha sentido avergonzada(o) o cohibida(o) por su aspecto físico?				
¿Se ha sentido físicamente menos atractiva(o) a consecuencia de la enfermedad o de su tratamiento?				
¿Se ha sentido descontenta(o) con su aspecto cuando está vestida(o)?				
¿Se ha sentido menos femenina/masculino como consecuencia de su enfermedad o tratamiento?				
¿Le resulta difícil mirarse cuando está desnuda(o)?				
¿Se ha sentido menos atractiva(o) sexualmente a consecuencia de su enfermedad o tratamiento?				
¿Ha evitado a alguien debido al modo en que se sentía en relación a su aspecto?				
¿Se ha sentido insatisfecha(o) con su cuerpo?				
