# Supplementary material for: The association of body image with quality of life, psychological assistance and social support in neurofibromatosis type 1 patients: a cross-sectional study
Source: Orphanet J Rare Dis. 2025 Jun 6;20:284. doi: 10.1186/s13023-025-03729-w (PMC12143036; doi:10.1186/s13023-025-03729-w)
Supplement: Supplementary file 2 — Supplementary material 2: EFA analysis: S-BIS modified – Validity and Reliability Requirements; The Exploratory Factorial Analysis of the S-BIS modified scale. [file 13023_2025_3729_MOESM2_ESM.docx]

| (α= 0.94) | EFA (1 Factor ) | | | Reliability Requirements | | | |
| --- | --- | --- | --- | --- | --- | --- | --- |
| Proportion of Variance ( 0.68 ) | Factorial Loads | Communalities | Uniqueness | Item – total correlation | Inter-item  correlation | Item-rest correlation | Cronbach’s  α |
| S-BIS Q1 | 0.90 | 0.81 | 0.19 | 0.86 | 0.67 | 0.81 | 0.93 |
| S-BIS Q2 | 0.89 | 0.79 | 0.21 | 0.91 | 0.65 | 0.87 | 0.93 |
| S-BIS Q3 | 0.85 | 0.71 | 0.29 | 0.85 | 0.67 | 0.80 | 0.93 |
| S-BIS Q4 | 0.83 | 0.69 | 0.31 | 0.75 | 0.70 | 0.69 | 0.94 |
| S-BIS Q5 | 0.83 | 0.68 | 0.32 | 0.85 | 0.67 | 0.79 | 0.93 |
| S-BIS Q6 | 0.82 | 0.67 | 0.33 | 0.87 | 0.67 | 0.82 | 0.93 |
| S-BIS Q7 | 0.73 | 0.54 | 0.46 | 0.77 | 0.69 | 0.71 | 0.94 |
| S-BIS Q9 | 0.71 | 0.50 | 0.50 | 0.90 | 0.66 | 0.86 | 0.93 |
| Validity Requirements | EFA | | | Established limits | | | |
| TLI | 0.95* | | | >0.90 | | | |
| RMSEA | 0.10 | | | < 0.08 | | | |
| RMSR | 0.04* | | | < 0.06 | | | |
| KMO | 0.94* | | | 0.90 - 1.00 (Marvelous) | | | |
| Bartlett | < 0.001** | | | <0.05 | | | |

**Additional File 2:** EFA analysis: S-BIS modified – Validity and Reliability Requirements

α, Cronbach’s alpha; EFA, exploratory factor analysis; TLI, Tucker-Lewis index, RMSEA, root mean square error of approximation; RMSR, root mean square residual; KMO, Kaiser-Meyer-Olkin test; Bartlett’s test of sphericity.
