# Supplementary material for: The association of body image with quality of life, psychological assistance and social support in neurofibromatosis type 1 patients: a cross-sectional study
Source: Orphanet J Rare Dis. 2025 Jun 6;20:284. doi: 10.1186/s13023-025-03729-w (PMC12143036; doi:10.1186/s13023-025-03729-w)
Supplement: Supplementary file 3 — Supplementary material 3: Correlations between independent and dependent variables; Correlations between independent and dependent variables [file 13023_2025_3729_MOESM3_ESM.docx]

| Variables | Ranges | M (SD) | 1. | 2. | 3. | 4. |
| --- | --- | --- | --- | --- | --- | --- |
| 1. S-BIS modified | 0 - 24 | 5.18 (6.25) | 1.00 |  |  |  |
| 2. Neurofibromas (Ad Hoc) | 0 - 3 | 1.96 (1.14) | 0.83 | 1.00 |  |  |
| 3. EQ-5D | 0.00 - 1.00 | 0.78 (0.21) | - 0.52 | - 0.37 | 1.00 |  |
| 4. EQ-VAS | 0 - 100 | 71.21 (26.60) | - 0.53 | - 0.41 | 0.61 | 1.00 |

**Additional File 3:** Correlations between independent and dependent variables

M, Means; SD, Standard deviations
