# Supplementary material for: The association of body image with quality of life, psychological assistance and social support in neurofibromatosis type 1 patients: a cross-sectional study
Source: Orphanet J Rare Dis. 2025 Jun 6;20:284. doi: 10.1186/s13023-025-03729-w (PMC12143036; doi:10.1186/s13023-025-03729-w)
Supplement: Supplementary file 4 — Supplementary material 4: Simple regressions with the independent variables and the EQ-5D subscales.; Simple regressions performed with the EQ-5D subscales [file 13023_2025_3729_MOESM4_ESM.docx]

**Additional File 4:** Simple regressions with the independent variables and the EQ-5D subscales.

| Variables | Total (n=205) | | | | | | |
| --- | --- | --- | --- | --- | --- | --- | --- |
|  | B | β | SE (B) | t value | Pr(>\|t\|) | 95% CI (B) | |
| S-BIS modified | | | | | | | |
| EQ-5D (Mobility) | 2.819 | 0.174 | 1.120 | 2.518 | 0.013* | 0.611 | 5.027 |
| EQ-5D (Self-care) | 3.522 | 0.171 | 1.425 | 2.472 | 0.014* | 0.713 | 6.332 |
| EQ-5D (Daily Activities) | 6.077 | 0.062 | 0.797 | 7.628 | <0.001*** | 4.506 | 7.648 |
| EQ-5D (Pain) | 4.129 | 0.414 | 0.637 | 6.482 | <0.001*** | 20.873 | 50.384 |
| EQ-5D (Anxiety/Depression) | 40.916 | 0.502 | 0.594 | 8.281 | <0.001*** | 3.745 | 6.086 |
| Neurofibromas (Ad Hoc) | | | | | | | |
| EQ-5D (Mobility) | 0.362 | 0.123 | 0.206 | 1.761 | 0.080 | -0.043 | 0.768 |
| EQ-5D (Self-care) | 0.259 | 0.069 | 0.263 | 0.985 | 326 | -0.259 | 0.777 |
| EQ-5D (Daily Activities) | 0.920 | 0.392 | 0.151 | 6.070 | <0.001*** | 0.621 | 1.218 |
| EQ-5D (Pain) | 0.591 | 0.325 | 0.121 | 4.901 | <0.001*** | 0.353 | 0.829 |
| EQ-5D (Anxiety/Depression) | 0.629 | 0.353 | 0.117 | 5.371 | <0.001*** | 0.398 | 0.860 |

B, regression coefficient; β, standardized regression coefficient; SE, standard error of B; t value of B; Pr (>|t|), Significance level: <0.05 (*), <0.01 (**), <0.001 (***); 95% CI, 95% confidence interval of B.
