# Supplementary material for: The association of body image with quality of life, psychological assistance and social support in neurofibromatosis type 1 patients: a cross-sectional study
Source: Orphanet J Rare Dis. 2025 Jun 6;20:284. doi: 10.1186/s13023-025-03729-w (PMC12143036; doi:10.1186/s13023-025-03729-w)
Supplement: Supplementary file 5 — Supplementary material 5: Correlations with Sources of Support; Correlations with the sources of support measures in the study [file 13023_2025_3729_MOESM5_ESM.docx]

**Additional File 5:** Correlations with Sources of Support

|  | | Sources of Support | | | | | | | | | | | |
| --- | --- | --- | --- | --- | --- | --- | --- | --- | --- | --- | --- | --- | --- |
|  |  | No / Social Networks | | No / Institutions | | No / Others | | No / Family | | No / Friends | | No / Partner | |
| Variable | Categories | ρ | Pr(>\|ρ\|) | ρ | Pr(>\|ρ\|) | ρ | Pr(>\|ρ\|) | ρ | Pr(>\|ρ\|) | ρ | Pr(>\|ρ\|) | ρ | Pr(>\|ρ\|) |
| Gender | Male/Female | -0.052 | 0.458 | 0.004 | 0.955 | 0.004 | 0.955 | 0.124 | 0.075 | 0.057 | 0.416 | -0.008 | 0.913 |
| Age | - | -0.065 | 0.352 | 0.093 | 0.186 | 0.045 | 0.526 | -0.239 | 0.001** | -0.238 | 0.001** | 0.199 | 0.004** |
| Age Groups | Adolescent/ Young Adult | -0.007 | 0.921 | -0.062 | 0.378 | -0.012 | 0.861 | 0.193 | 0.005** | 0.177 | 0.011* | -0.183 | 0.009** |
|  | Adolescent/ Adult | 0.079 | 0.260 | 0.089 | 0.203 | -0.010 | 0.885 | -0.059 | 0.401 | 0.018 | 0.796 | 0.107 | 0.128 |
|  | Adolescent/ Middle Aged | -0.064 | 0.364 | -0.054 | 0.442 | 0.062 | 0.378 | -0.186 | 0.008** | -0.219 | 0.002** | 0.110 | 0.117 |
|  | Adolescent/ Aged | -0.023 | 0.744 | 0.091 | 0.195 | -0.040 | 0.566 | -0.056 | 0.424 | -0.094 | 0.182 | -0.023 | 0.747 |
| Marital Status | Single/ Married | -0.074 | 0.293 | 0.085 | 0.225 | 0.031 | 0.654 | -0.143 | 0.041* | -0.253 | <0.001*** | 0.239 | <0.001*** |
|  | Single/ Divorced | -0.025 | 0.727 | -0.043 | 0.539 | -0.043 | 0.539 | 0.019 | 0.788 | 0.047 | 0.499 | 0.031 | 0.662 |
|  | Single/ Widowed | -0.009 | 0.903 | -0.015 | 0.831 | -0.015 | 0.831 | -0.109 | 0.120 | -0.055 | 0.430 | -0.034 | 0.629 |
| Education | No-studies/ Primary school | -0.047 | 0.499 | 0.198 | 0.004** | -0.013 | 0.853 | 0.060 | 0.390 | -0.012 | 0.864 | 0.105 | 0.133 |
|  | No-studies/ Middle school | -0.058 | 0.408 | -0.102 | 0.145 | -0.041 | 0.560 | -0.052 | 0.461 | -0.068 | 0.331 | 0.025 | 0.726 |
|  | No-studies/ High school | 0.072 | 0.306 | 0.028 | 0.688 | 0.028 | 0.688 | -0.004 | 0.957 | 0.019 | 0.782 | -0.098 | 0.161 |
|  | No-studies/ University | 0.014 | 0.846 | -0.082 | 0.244 | 0.024 | 0.733 | 0.047 | 0.503 | 0.081 | 0.247 | -0.001 | 0.989 |
| Employment | Unemployed/ Employed | -0.046 | 0.512 | -0.081 | 0.249 | 0.014 | 0.838 | -0.165 | 0.018* | -0.025 | 0.725 | 0.116 | 0.099 |
|  | Unemployed/ Paid leave | -0.030 | 0.665 | 0.048 | 0.494 | -0.053 | 0.447 | 0.069 | 0.325 | 0.016 | 0.820 | 0.091 | 0.195 |
|  | Unemployed/ Retired | -0.015 | 0.833 | -0.026 | 0.710 | -0.026 | 0.710 | -0.100 | 0.153 | -0.096 | 0.169 | 0.044 | 0.527 |
| Skin Severity Index | - | -0.104 | 0.139 | 0.047 | 0.501 | 0.009 | 0.893 | -0.115 | 0.101 | -0.084 | 0.231 | -0.044 | 0.534 |
|  | Low Severity/ Medium Severity | -0.072 | 0.305 | 0.037 | 0.602 | -0.018 | 0.800 | -0.159 | 0.023* | -0.147 | 0.035* | 0.026 | 0.711 |
|  | Low Severity/ High Severity | -0.057 | 0.415 | 0.023 | 0.741 | 0.023 | 0.741 | -0.005 | 0.946 | 0.019 | 0.783 | -0.066 | 0.348 |
| Psychological Assistance | No/ Yes | 0.097 | 0.168 | 0.066 | 0.345 | 0.170 | 0.015* | 0.190 | 0.006** | 0.155 | 0.026* | -0.022 | 0.760 |
| Sources of Support | No / Social Networks | - | - | -0.026 | 0.710 | 0.117 | 0.095 | 0.078 | 0.264 | 0.070 | 0.316 | -0.059 | 0.400 |
|  | No / Institutions | -0.026 | 0.710 | - | - | -0.026 | 0.710 | -0.019 | 0.785 | 0.075 | 0.286 | 0.017 | 0.804 |
|  | No / Others | -0.026 | 0.710 | -0.046 | 0.513 | - | - | 0.086 | 0.223 | -0.023 | 0.744 | -0.043 | 0.539 |
|  | No / Family | 0.078 | 0.264 | -0.019 | 0.785 | 0.086 | 0.223 | - | - | 0.465 | <0.001*** | 0.175 | 0.012* |
|  | No / Friends | 0.070 | 0.316 | 0.075 | 0.286 | -0.023 | 0.744 | 0.465 | <0.001*** | - | - | 0.255 | <0.001*** |
|  | No / Partner | -0.059 | 0.400 | 0.017 | 0.804 | -0.043 | 0.539 | 0.175 | 0.012* | 0.255 | <0.001*** | - | - |
| EQ-5D | - | 0.026 | 0.708 | -0.086 | 0.222 | -0.058 | 0.411 | -0.169 | 0.015* | 0.003 | 0.965 | -0.063 | 0.370 |
| EQ-VAS | - | 0.021 | 0.769 | -0.090 | 0.198 | -0.069 | 0.325 | -0.078 | 0.267 | -0.001 | 0.991 | -0.102 | 0.145 |
| S-BIS modified | - | -0.046 | 0.517 | 0.118 | 0.093 | 0.058 | 0.412 | 0.080 | 0.252 | 0.074 | 0.291 | 0.069 | 0.326 |
| Neurofibromas (Ad Hoc) | - | -0.013 | 0.858 | 0.057 | 0.419 | -0.002 | 0.973 | -0.002 | 0.979 | 0.017 | 0.808 | 0.065 | 0.356 |

ρ, Spearman’s coefficient; Pr (>|ρ|), Significance level: <0.05 (*), <0.01 (**), <0.001 (***)
